# 

**Published:** 1922-12

**Authors:** 


### RECENT APPOINTMENTS.

### Public Health.

Mr. R. H. Vercoe, M.R.C.S., L.R.C.P.(Lond.), D.P.H. (Oxon.), formerly Assistant Medical Officer of Health at Colchester, Ealing and Southall, to be Medical Officer of Health and School Medical Officer for the Borough of Chelmsford.

Miss Freda M. Andrews, formerly house surgeon at the Royal Dental Hospital, and school dentist to the Portsmouth Education Committee, to be Dental Officer to the County Council of Middlesex.

The Lord Chancellor has appointed Lieutenant-Colonel Nathan Raw, C.M.G., M.D., to be a Medical Visitor of Lunatics, so found by inquisition.

Dr. William Stott, M.B., B.S.(Hons.), to be Medical Officer for Venereal Diseases for Burnley.

Dr. D. H. Geffen, M.R.C.S., M.B.B.S.(Hons.), L.R.C.P., D.P.H., M.D., to be Assistant Tuberculosis Officer for Poplar.

Miss G. D. Maclaren, M.B., to be Woman Assistant Medical Officer to the County of the parts of Lindsey (Lincs.).

### Institutional.

Shropshire Orthopædic Hospital and Agnes Hunt Surgical Home, Oswestry (late Shropshire Surgical Home). Miss A. G. Hunt, R.R.C., the founder of the hospital, has been appointed matron in the place of Miss M. Beesley, resigned.

---

### GOVERNMENT PUBLICATIONS.

Copies can be purchased through any bookseller or direct from H.M. Stationery Office, at Imperial House, Kingsway, W.C.2; 28 Abingdon Street, S.W.1; 37 Peter Street, Manchester; 23 Forth Street, Edinburgh; and 1 St. Andrew's Crescent, Cardiff. The prices quoted include postage.]

STATIONERY OFFICE PUBLICATIONS.

Board of Education:—
- Health of the School Child. Annual Report of the Chief Medical Officer for 1921. 1s. 8d.

Ministry of Health:—
- Byelaws. Model: XVI. Offensive Trades. 1s. 7d.
- Blindness. Causes and Prevention. Departmental Committee. Final Report. 4s. 2½d.
- Smallpox: Memo. 71/Med. Memorandum on the steps specially requisite to be taken by Boards of Guardians. 1½d.
- Memo. 71a/Med. Memorandum on the steps requisite to be taken by Sanitary Authorities. 1½d.

---

ON December 14, 15 and 16 Hyde Park Corner will be the scene of a large Christmas Market organised by the Health and Empire Committee, whose object is to educate the public on personal hygiene, realising as it does that ill-health undermines efficiency. At present this work is being carried out by means of lectures, literature and cinema films, which are shown in towns and villages. This naturally involves enormous expense, and since health is a matter which affects all, the Organising Committee, of which the Countess of Shaftesbury is chairman, hopes that all will give their support. Money is not asked for, but gifts of useful produce suitable for a Christmas Market. Everything received will be allocated to its appropriate section—the farm, the garden, the nursery, the store cupboard, the wardrobe or the home—and will be sold at current market prices.

Not only will donors have the pleasure of knowing that they are doing good by their gifts, they may also be fortunate enough to win a prize, as there are to be competitions in each section—*e.g.*, prizes of £3, £2, £1, will be given for the best pound of butter, and so on. Another interesting feature of the market will be the Empire Tea Room, under the patronage of the Official Representatives of the Dominions and the Crown Colonies, whilst there is to be a special Overseas Section, displaying all gifts from Overseas. The Crown property of No. 145, Piccadilly, W. 1, has very generously been lent free of charge, and the Market offices will be open there from Monday, November 6. Further particulars may be obtained from the secretary at that address.

